# Where Molecules Meet Mucus: Mutanofactins in the Oral Microbiome

**DOI:** 10.1021/acscentsci.5c00546

**Published:** 2025-04-07

**Authors:** McKenna Loop Yao, Wenjun Zhang

**Affiliations:** Department of Chemical and Biomolecular Engineering, University of California, Berkeley, California 94720, United States

Oral health is increasingly being recognized as a critical indicator of broader systemic health.^1^ Emerging research has linked the composition of the oral microbiome to several systemic diseases, including cardiovascular disease, Alzheimer’s disease, diabetes, and some cancers.^1,2^ Beyond these conditions, common oral conditions such as cavities (dental caries), periodontitis, and gingivitis contribute to an enormous global economic burden exceeding $350 billion annually.^3^ At the heart of oral health lies a complex relationship between the host and the oral microbiome, driven predominantly by bacterial biofilms—the primary mode by which microbes colonize oral surfaces.^4^ Understanding biofilm dynamics could yield targeted interventions beyond conventional brushing and flossing. In this issue of *ACS Central Science*, Reimhult, Schäffer, Carreira, and co-workers chemically synthesized a group of bioactive small molecules naturally produced by oral bacteria and revealed how these metabolites could potentially influence biofilm dynamics and contribute to disease through a unique biophysical mechanism.^2^ Their work represents an important advancement toward elucidating the molecular complexity of biofilms in the oral microbiome, underscoring the potential of microbiome-derived natural products to advance oral health interventions.

Biofilm formation is central to oral disease development.^4^ Initially, Gram-positive facultative anaerobes (e.g., *Streptococcus mutans* or *Streptococcus sanguinis*) adhere to teeth, producing acid and extracellular polymeric substances.^5^ As the biofilm matures and oxygen decreases, strict anaerobes begin to dominate, causing a local bacterial imbalance and eventual enamel demineralization, caries formation, and periodontal disease.^4^ Although the molecular foundations of biofilm formation and their link to oral diseases have been heavily investigated, emerging discoveries within the oral microbiome strongly indicate that our understanding of biofilm formation remains significantly incomplete. For instance, in 2021, Li and co-workers discovered mutanofactins, which are produced by a subset of *S. mutans* clinical isolates and promote biofilm formation of *S. mutans* (Figure 1).^6^ This was the first example of an oral pathogen-derived bioactive metabolite directly involved in biofilm formation, beyond the typical signaling function of small molecules. This discovery highlighted the underexplored role of small, bioactive molecules produced by microbes for complex cell–cell and cell-host interactions in shaping human oral health.^7^

**Figure 1:**
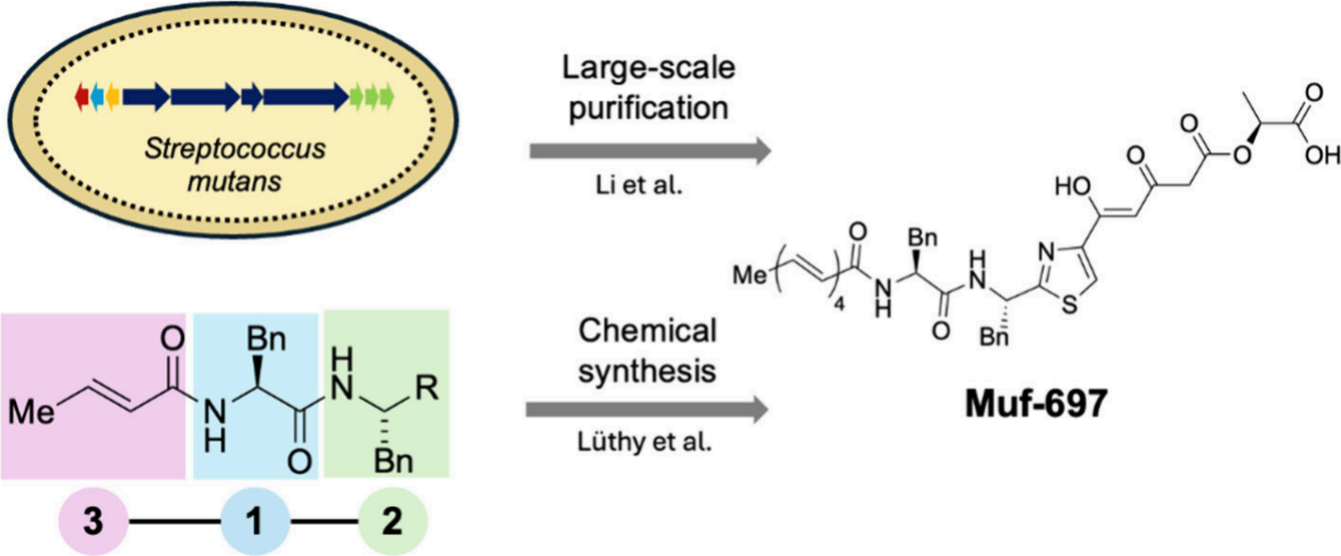
Mutanofactin-697 was produced via large-scale microbial production by Li et al. and chemically synthesized by Lüthy et al. through a 3-step chemical synthesis scheme. The mutanofactin gene cluster from *Streptococcus mutans* is shown as arrows within the *S. mutans* cell.

The initial mechanistic characterization of a major mutanofactin metabolite, Muf-697, suggested that mutanofactin-induced biofilm formation mainly resulted from its surfactant-like lipopeptide structure influencing cell hydrophobicity and promoting cell adherence.^6^ However, key questions remained about the role of mutanofactins within the broader oral microbiome and the precise mechanisms underlying their biofilm-promoting activity. The current study by Lüthy et al. provides deeper insight into the bioactivity of mutanofactins through total chemical synthesis, rigorous bioactivity testing, and a biophysical characterization of a unique interaction between mutanofactins and a host defense mechanism.^2^ Specifically, Lüthy and co-workers successfully chemically synthesized Muf-697 and its four chemical variants, a notable achievement as mutanofactins are complex nonribosomal peptide and polyketide hybrid lipopeptides^8^ with low production titers from *S. mutans* (Figure 1). The complete chemical synthesis of mutanofactins allowed the authors to rigorously test activities of these metabolites and to probe molecular mechanisms. Interestingly, adding Muf-697 to a wildtype mutanofactin producer decreased biofilm formation, suggesting a possible cytotoxic effect of mutanofactins and a complex self-regulatory biosynthesis scheme. Lüthy et al. also showed mutanofactins have selective, species-specific effects on other oral bacteria: early colonizers like *S. gordonii and S. oralis* exhibited increased biofilm formation, while species such as *Fusobacterium nucleatum* and *Veillonella dispar* remained mostly unaffected (Figure 2A).

**Figure 2:**
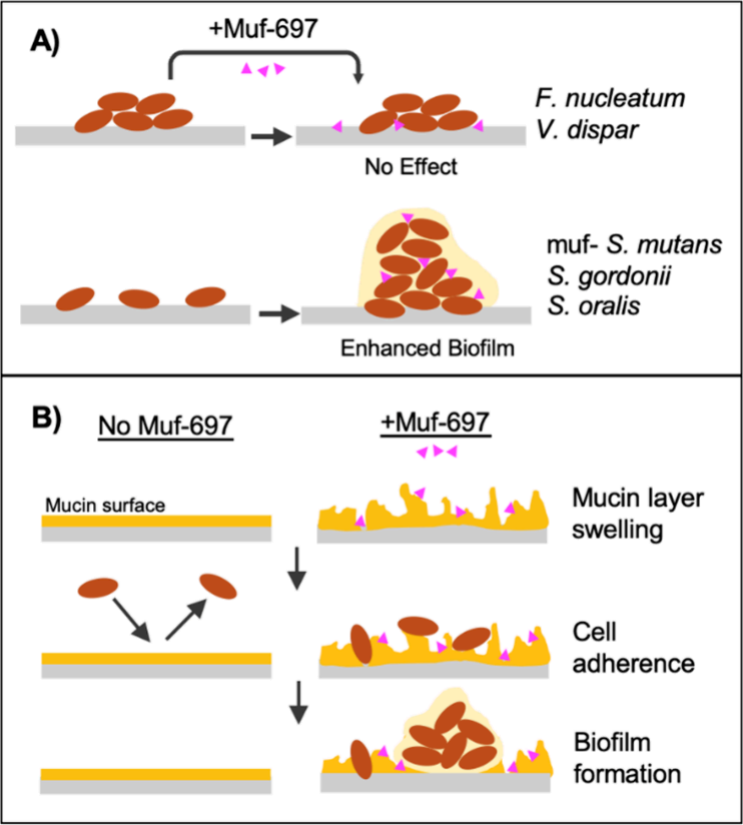
A) Effect of mutanofactin-697 on biofilm formation in various oral microbial strains. B) Effect of mutanofactin-697 on a mucin-coated surface, quantified via quartz crystal microbalance (QCM-D) and atomic force microscopy (AFM). The mucin layer height increases and “swells” upon addition of Muf-697, which facilitates the binding of *S. mutans* to the surface. Pink triangles represent Muf-697, and orange ovals represent microbial cells.

More intriguingly, the authors revealed for the first time the interaction of Muf-697 and mucins using quartz crystal microbalance (QCM-D) and atomic force microscopy (AFM, Figure 2B). Mucins are heavily glycosylated proteins produced by the host and are a major component of oral mucus that modulate bacterial adhesion and community structure via their glycan substrates.^9^ Mucins have been shown to support cooperative metabolism and niche partitioning among beneficial oral microbes while reducing biofilm density and preventing the overpopulation of pathogenic species. Muf-697 was found to alter the structural properties of the mucin layer, which allowed *S. mutans* to adhere onto the otherwise protective layer. *S. mutans* mutants lacking mutanofactin production showed minimal adherence to a mucin-coated surface, whereas readdition of Muf-697 restored adherence and subsequent biofilm formation.

Although these findings compellingly establish the interaction of Muf-697 with mucins, critical questions remain about its precise chemical mechanism and broader microbiome implications. Future studies could investigate how Muf-697 chemically interacts with mucins—can these interactions be directly visualized or structurally characterized? As a distinct new mode of action—beyond the known mechanisms of modulating hydrophobicity and interacting with eDNA—how significant is this new mechanism of Muf-697 in its natural ecological niche? Are there any relationships between the mucin interaction and the observed differential impact of Muf-697 on biofilm formation of cohabitating oral bacteria? What is the major role of mutanofactins within a dynamic and complex oral microbial community?

Though there is still much to understand, Reimhult, Schäffer, Carreira, and co-workers beautifully intertwined organic synthesis with microbiology and biophysics to uncover a unique interaction of mutanofactins with a host mucin defense mechanism. Their findings underscore the significant, yet previously underappreciated, role specialized metabolites play in shaping microbial dynamics within—and potentially beyond—the oral cavity. These findings position specialized microbial metabolism as an untapped resource for breakthrough interventions in oral health and their systemic consequences.

## References

[ref1] Peng X.; Cheng L.; You Y.; Tang C.; Ren B.; Li Y.; Xu X.; Zhou X. Oral Microbiota in Human Systematic Diseases. Int. J. Oral Sci. 2022, 14 (1), 1–11. 10.1038/s41368-022-00163-7. 35236828 PMC8891310

[ref2] Luthy L.; Thies L. G. S.; Beitl K. N.; Hansen M.; McManus J.; Afzal M.; Schrangl L.; Bloch S.; Subbiahdoss G.; Reimhult E.; Schaffer C.; Carreira E. M. Synthesis, Microbiology, and Biophysical Characterization of Mutanofactins from the Human Oral Microbiome. ACS Cent. Sci. 2025, 10.1021/acscentsci.4c02184. PMC12022917 40290153

[ref3] Righolt A. J.; Jevdjevic M.; Marcenes W.; Listl S. Global-, Regional-, and Country-Level Economic Impacts of Dental Diseases in 2015. J. Dent. Res. 2018, 97 (5), 501–507. 10.1177/0022034517750572. 29342371

[ref4] Sharma S.; Mohler J.; Mahajan S. D.; Schwartz S. A.; Bruggemann L.; Aalinkeel R. Microbial Biofilm: A Review on Formation, Infection, Antibiotic Resistance, Control Measures, and Innovative Treatment. Microorganisms 2023, 11 (6), 1614 10.3390/microorganisms11061614. 37375116 PMC10305407

[ref5] Lemos J. A.; Palmer S. R.; Zeng L.; Wen Z. T.; Kajfasz J. K.; Freires I. A.; Abranches J.; Brady L. J. The Biology of Streptococcus Mutans. Microbiol. Spectr. 2019. 10.1128/microbiolspec.GPP3-0051-2018. PMC6615571 30657107

[ref6] Li Z.-R.; Sun J.; Du Y.; Pan A.; Zeng L.; Maboudian R.; Burne R. A.; Qian P.-Y.; Zhang W. Mutanofactin Promotes Adhesion and Biofilm Formation of Cariogenic Streptococcus Mutans. Nat. Chem. Biol. 2021, 17 (5), 576–584. 10.1038/s41589-021-00745-2. 33664521

[ref7] Aleti G.; Baker J. L.; Tang X.; Alvarez R.; Dinis M.; Tran N. C.; Melnik A. V.; Zhong C.; Ernst M.; Dorrestein P. C.; Edlund A. Identification of the Bacterial Biosynthetic Gene Clusters of the Oral Microbiome Illuminates the Unexplored Social Language of Bacteria during Health and Disease. mBio 2019, 10 (2), e00321–19 10.1128/mBio.00321-19. 30992349 PMC6469967

[ref8] Fischbach M. A.; Walsh C. T. Assembly-Line Enzymology for Polyketide and Nonribosomal Peptide Antibiotics: Logic, Machinery, and Mechanisms. Chem. Rev. 2006, 106 (8), 3468–3496. 10.1021/cr0503097. 16895337

[ref9] Wu C. M.; Wheeler K. M.; Cárcamo-Oyarce G.; Aoki K.; McShane A.; Datta S. S.; Mark Welch J. L.; Tiemeyer M.; Griffen A. L.; Ribbeck K. Mucin Glycans Drive Oral Microbial Community Composition and Function. Npj Biofilms Microbiomes 2023, 9 (1), 1–14. 10.1038/s41522-023-00378-4. 36959210 PMC10036478

